# Evaluation of the deliverability of dynamic conformal arc therapy (DCAT) by gantry wobble and its influence on dose

**DOI:** 10.1038/s41598-024-57644-4

**Published:** 2024-03-26

**Authors:** Changhwan Kim, Hojae Kim, Dongmin Jung, Heesoo Kim, Yeonok Park, Min Cheol Han, Chae-Seon Hong, Hojin Kim, Ho Lee, Jinsil Sung, Dong Wook Kim, Jin Sung Kim

**Affiliations:** 1 Department of Radiation Oncology, Yonsei Cancer Center, Yonsei University College of Medicine, Seoul, South Korea; 2 Department of Radiation Oncology, Yonsei Cancer Center, Seoul, South Korea; 3 Department of Radiation Oncology, National Cancer Center, Proton Therapy Center, Goyang, South Korea

**Keywords:** Biomedical engineering, Radiotherapy

## Abstract

We aimed to investigate the deliverability of dynamic conformal arc therapy (DCAT) by gantry wobble owing to the intrinsic inter-segment break of the Elekta linear accelerator (LINAC) and its adverse influence on the dose to the patient. The deliverability of DCAT was evaluated according to the plan parameters, which affect the gantry rotation speed and resultant positional inaccuracies; the deliverability according to the number of control points and dose rates was investigated by using treatment machine log files and dosimetry devices, respectively. A non-negligible degradation in DCAT deliverability due to gantry wobble was observed in both the treatment machine log files and dosimetry devices. The resulting dose-delivery error occurred below a certain number of control points or above a certain dose rate. Dose simulations in the patient domain showed a similar impact on deteriorated deliverability. For targets located primarily in the isocenter, the dose differences were negligible, whereas for organs at risk located mainly off-isocenter, the dose differences were significant up to − 8.77%. To ensure safe and accurate radiotherapy, optimal plan parameters should be selected, and gantry angle-specific validations should be conducted before treatment.

## Introduction

Stereotactic body radiation therapy (SBRT) has emerged as a promising radiotherapy regimen, particularly for small tumors. In SBRT, a high fractional dose can be delivered to tumors in a hypofractionated manner, which allows for better local control while maintaining relatively low toxicity^1–4^. Thus, SBRT requires intensive and conformal dose delivery until the target volume is reached while sparing adjacent surrounding tissues with a steep dose fall-off.

To achieve rapid dose gradient, complex modulation of treatment system related parameters, including gantry rotation speed, dose rates, and multileaf collimator (MLC) movements, is required. Among the various treatment delivery techniques, arc therapy-based approaches are preferred because of their sufficient degrees of freedom in achieving the abovementioned conditions with reduced treatment time. Volumetric-modulated arc therapy (VMAT) and dynamic conformal arc therapy (DCAT) are representative methods.

VMAT employs a gantry rotation of up to 360° with modulation of the gantry rotation speed, dose rates, and MLC movements. However, DCAT rarely employs MLC modulation, except for shaping the target to deliver a conformal dose. DCAT, which is a simplified version of VMAT, has several advantages in terms of clinical applications. By reducing plan complexity, the mechanical burden on the treatment machine is decreased, so plan deliverability can be relatively improved. Another advantage is that a clinical plan can be efficiently generated with a small calculation burden on the treatment planning system (TPS). In addition, the decreased plan complexity leads to a reduction in the total number of monitor units (MUs) needed to deliver the same prescribed dose, thus reducing the treatment time accordingly. DCAT can also minimize the interplay effect between treatment machine parameters and moving tumors because its field shape encompasses the entire target volume at all beam angles^5,6^.

Although DCAT has various clinical merits, its plan quality and consequent dose distribution may not be superior to those of VMAT, owing to its relatively lower degrees of freedom. Because DCAT has a small modulation of MLC movement, it is difficult to generate a steep dose gradient; thus, the performance of OAR sparing may be insufficient compared with that of VMAT plans. Therefore, because of the clinical advantages and disadvantages of DCAT, its clinical suitability for various treatment sites has been investigated, and it has been reported that the plan quality thereof is comparable to VMAT for tumors with a small target size^7–10^. Therefore, various studies have been conducted that employ DCAT as the treatment delivery technique for lung and liver SBRT cases to maximize the clinical advantages of DCAT. Regarding dose, the DCAT technique for lung and liver SBRT is an efficient alternative to VMAT.

Various commercially available clinical TPSs support the DCAT technique. At our institute, we utilize Monaco version 5.51 (Elekta AB, Stockholm, Sweden), which employs Monte Carlo-based dose calculations, to create DCAT-based radiation therapy plans. The DCAT plans are then delivered by using an Elekta Versa HD linear accelerator. The Elekta LINAC has an intrinsic inter-segment break for every 1,000 MU. Therefore, when delivering a high dose per fraction in SBRT treatment with a large number of MU, it is inevitable to have one or more inter-segment breaks. Depending on the condition of the machine, when the gantry rotates and stops at an inter-segment breakpoint while delivering radiation, it may be difficult to stop at the exact position owing to inertia. This can result in positional inaccuracies in the gantry angle in the form of gantry wobble. Moreover, this positional error is more likely to occur when the gantry rotation speed is higher and inertia increases.

In the arc-therapy based approaches, the mechanical and relevant parameters of MLC speed, dose rate, and gantry rotation speed are chosen conservatively to avoid stressing the LINAC system^11–13^. Therefore, from this perspective, DCAT-based plans have less modulation compared to VMAT, resulting in fewer control points and mechanical constraints. Consequently, there is a higher potential for increased gantry rotation speed, leading to a higher likelihood of positional inaccuracies at the inter-segment breakpoints. This may affect the delivery of the treatment plan and lead to errors in MU delivery. Furthermore, because the MU per control point is larger than in the VMAT plan, the number of MU delivery errors caused by the gantry wobble may be greater.

The deliverability of DCAT may, therefore, directly affect the dose delivered to the patient. We aimed to investigate the impact of gantry wobble caused by inter-segment breaks in the Elekta LINAC on the deliverability of DCAT plans. Specifically, deliverability was evaluated according to the plan parameters of the DCAT that affect the gantry rotation speed and resultant positional inaccuracies. We also investigated the adverse influence of degraded deliverability on dose discrepancies between the actual and planned doses delivered to the patient.

## Methods

### Evaluation of DCAT deliverability according to the plan parameters

The number of control points is a complex plan parameter that is determined by various factors, including MLC speed, gantry speed, dose rate, and MU. As the number of control points increases, the gantry rotation speed may decrease to match the additional time required for adjustment and movement between two subsequent control points. Furthermore, even in the case of DCAT, where the additional time is negligible owing to less MLC modulation, if the number of control points is large enough that the gantry spacing between two subsequent control points is small, the arc length of the gantry rotation for each becomes short. Consequently, the gantry may move to the next control point before reaching the maximum gantry acceleration per control points, potentially limiting the use of the maximum gantry rotation speed. By contrast, a small number of control points may lead to faster gantry rotations^14,15^. In any case, the number of control points in the DCAT plan is one of the main factors affecting the gantry rotation speed; therefore a non-negligible gantry wobble can occur at the intrinsic inter-segment breakpoint of the Elekta LINAC, especially at high rotational speeds. When creating a DCAT plan with Monaco, the number of control points is determined by adjusting the ‘Inc’ parameter which is the increment of the beam, thus controls the angular increment of the beam in TPS. Basically, beam segments (equivalent to the number control points -1) are calculated by dividing the total arc degree into ‘Inc’, but in the case of DCAT, ‘Inc’ is applied by half, as fixed by default. To investigate the DCAT’s deliverability according to the number of control points, the angular increments were set as 15°, 7.5°, 5°, 4°, 2.5°, and 1.5°. Plans with 25, 49, 73, 91, 145, and 241 control points were prepared. These plans were generated for the two X-ray energies that are primarily selected for DCAT at our institution: unflattened 6 MV (6MV-FFF (Flattening Filter Free)) and unflattened 10 MV (10 MV-FFF).

Similarly, dose rate is another fact that can affect gantry rotation speed. A low dose rate results in a longer time to deliver the MU assigned to each control point, which hinders to fully use of the maximum gantry rotation speed. However, at a high dose rate, the MU assigned to each control point can be delivered in a shorter time, allowing the gantry to rotate much faster. In other words, a higher dose rate enables the effective utilization of the maximum gantry rotation speed. Thus, in such situations, the occurrence of inter-segment breaks may lead to a gantry wobble that is significant enough to adversely affect the deliverability of the DCAT plan owing to increased inertia. Because the dose rate variation within the plan is not significant in DCAT, instead of individually adjusting the dose rate in the plan, we modified the X-ray energy for each plan to enable the use of the maximum dose rate for each energy. Plans with 25 control points were employed and we selected three different energies: 6 MV, 6 MV-FFF, and 10 MV-FFF. The corresponding maximum dose rates for each energy of the Elekta LINAC were 600 MU/min, 1400 MU/min, and 1800 MU/min.

The impact of the control points and dose rate on the deliverability of DCAT was investigated using the ArcCHECK phantom (SNC, Melbourne, FL, USA), a cylindrical acrylic phantom with a semi three-dimensional diode array. Its diameter is 21 cm and it contains 1,386 diode detectors arranged in a helical pattern, with 10 mm spacing, which translates to 7 mm spacing when viewed from the beam's perspective. Its shape and array arrangement allow for the measurement and analysis of beam fluence, depending on the gantry angle. Therefore, it was selected as the measurement device for this study because it can evaluate the gantry positional error and relevant MU errors at the point where the inter-segment break occurs. In addition to the study based on the dosimetry device, we concurrently examined the treatment machine log files to assess DCAT deliverability based on the number of control points and dose rate. Detailed descriptions are provided in Supplementary Material B.

### Evaluation of dosimetric impact according to the deliverability

To evaluate the dosimetric impact of delivery errors on the patient that may occur at inter-segment breaks, actual dose measurements were performed by using Octavius 1000 SRS (PTW, Freiburg, Germany), a 2D liquid-filled ionization chamber detector array typically employed at our institute for patient-specific pretreatment quality assurance (QA). It contains 977 liquid-filled ion chambers arranged over an area of $11\times 11 {{\text{cm}}}^{2}$, with 2.5 mm center-to-center spacing. For the measurement, the same experimental setup described in our previous study^16^ was used, and the measurement depth was set to 5 cm with a source-to-surface distance of 95 cm, which is equivalent to the reference point of measurement set at 100 cm from the source. No additional 4D rotational unit with a LINAC gantry was utilized in this work. The integrated dose on the 2D array was obtained according to the number of control points and dose rate. The measured dose was then compared to that calculated from the TPS using gamma analysis. For detailed analysis, local gamma evaluation with 10% low-dose threshold (the percentage of maximum dose below which data were excluded) was conducted using two individual criteria: 3%/3 mm and 1%/1 mm, respectively.

In addition to measurement-based evaluation, the effect of delivery errors on patient dose was investigated through dose recalculation with a modified RT-plan that changed MU weights according to detected delivery errors. As described in Supplemental Material B, delivery errors at the inter-segment breakpoints were perceived from the machine log files. The ‘Cumulative meterset weight’ for each control point in RT-plan was modified accordingly to implement simulated errors at a level similar to the measured delivery errors. To recalculate the dose of the modified plan, Mobius3D (Varian Medical Systems, Inc., CA, USA), a secondary dose check software, was utilized for this study. Detailed descriptions are provided in Supplementary Material D.

### Experimental conditions

Two patients who underwent SBRT with the DCAT plan at our institute between September 2021 and August 2022 were included as follow: one case in which the internal mammary lymph node was treated using a fractional dose of 8 Gy for five fractions with 6 MV-FFF (case 1), and another case in which the liver was treated with a fractional dose of 12 Gy for four fractions using 10 MV-FFF (case 2). Plans with different settings for the number of control points and dose rates were prepared as described in Methods section above. The same optimization criteria applied to create the plans for actual treatment were used when generating the plans, ensuring a similar level of plan quality regardless of the differences in the plan parameters. The dose distribution of the reference plans used in this study are provided in Supplementary Material A. The patient characteristics, including plan parameters, are summarized in Table 1. This retrospective study was approved by the Institutional Review Board (IRB) of Yonsei University Hospital (approval number: 4-2023-0869). All data were fully anonymized before the investigators accessed them. Informed consent was waived by IRB of Yonsei University Hospital. All relevant guidelines and regulations were followed.

**Table 1:** Patient characteristics

Case	Site	Fractional dose (Gy)	Fx	Energy	Gantry rotation angles	Number of control points					
						25	49	73	91	145	241
						MU					
1	Internal mammary lymph node	8	5	6 MV-FFF	360˚ (− 180˚ to 180˚)	1374	1378	1374	1378	1370	1375
2	Liver	12	4	10 MV-FFF		2473	2490	2395	2415	2413	2369

*Fx* fraction; *MU* monitor unit.

### Ethic statement

This retrospective study was approved by the Institutional Review Board (IRB) of Yonsei University Hospital (approval number: 4-2023-0869). All data were fully anonymized before the investigators accessed them. Informed consent was waived by IRB of Yonsei University Hospital. All relevant guidelines and regulations were followed.

## Results

### Evaluation of DCAT deliverability according to the plan parameters

**Figure 1:**
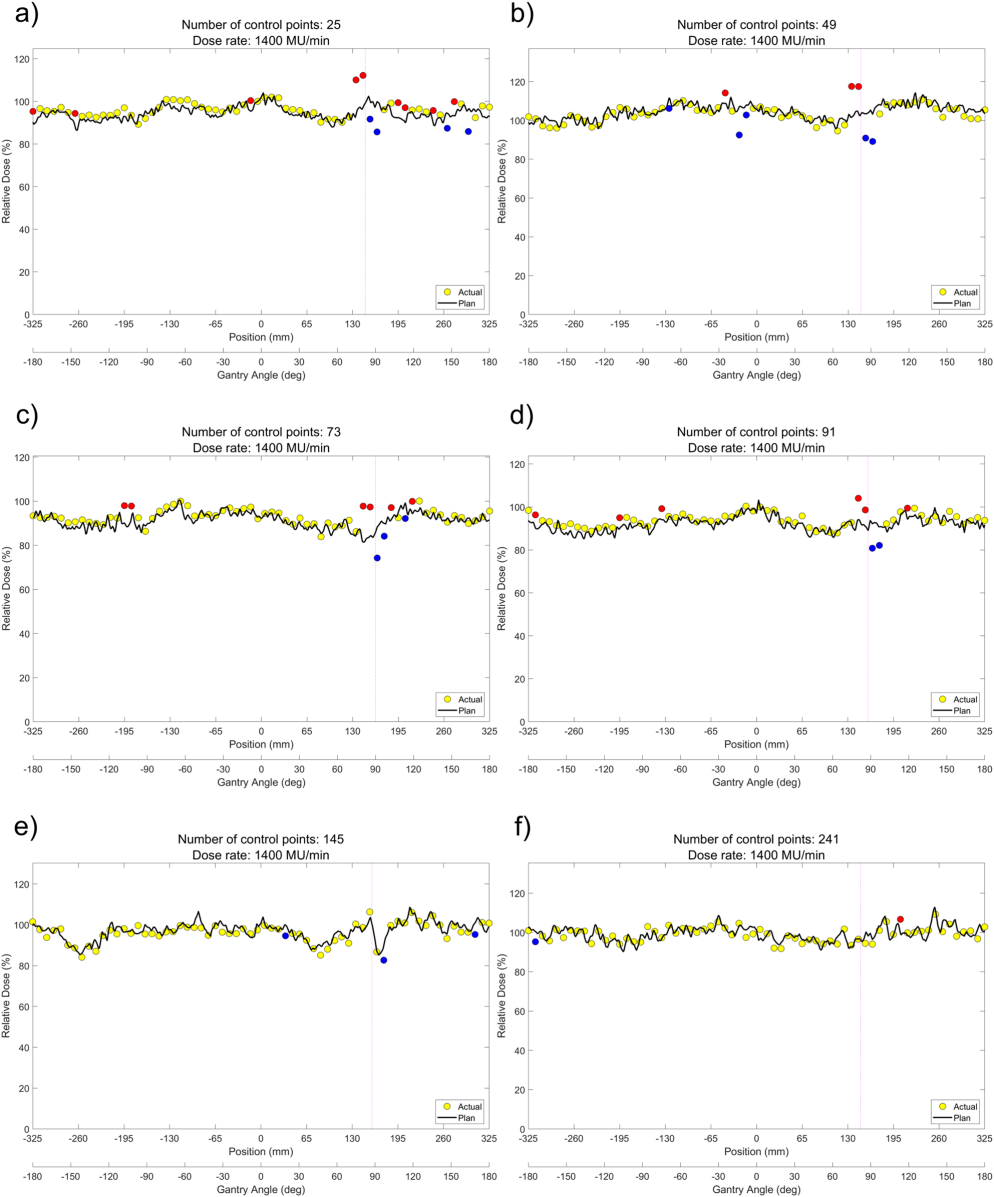
ArcCHECK measurements of 6 MV-FFF DCAT plans with different numbers of control points. (**a**) 25, (**b**) 49, (**c**) 73, (**d**) 91, (**e**) 145, and (**f**) 241.

**Figure 2:**
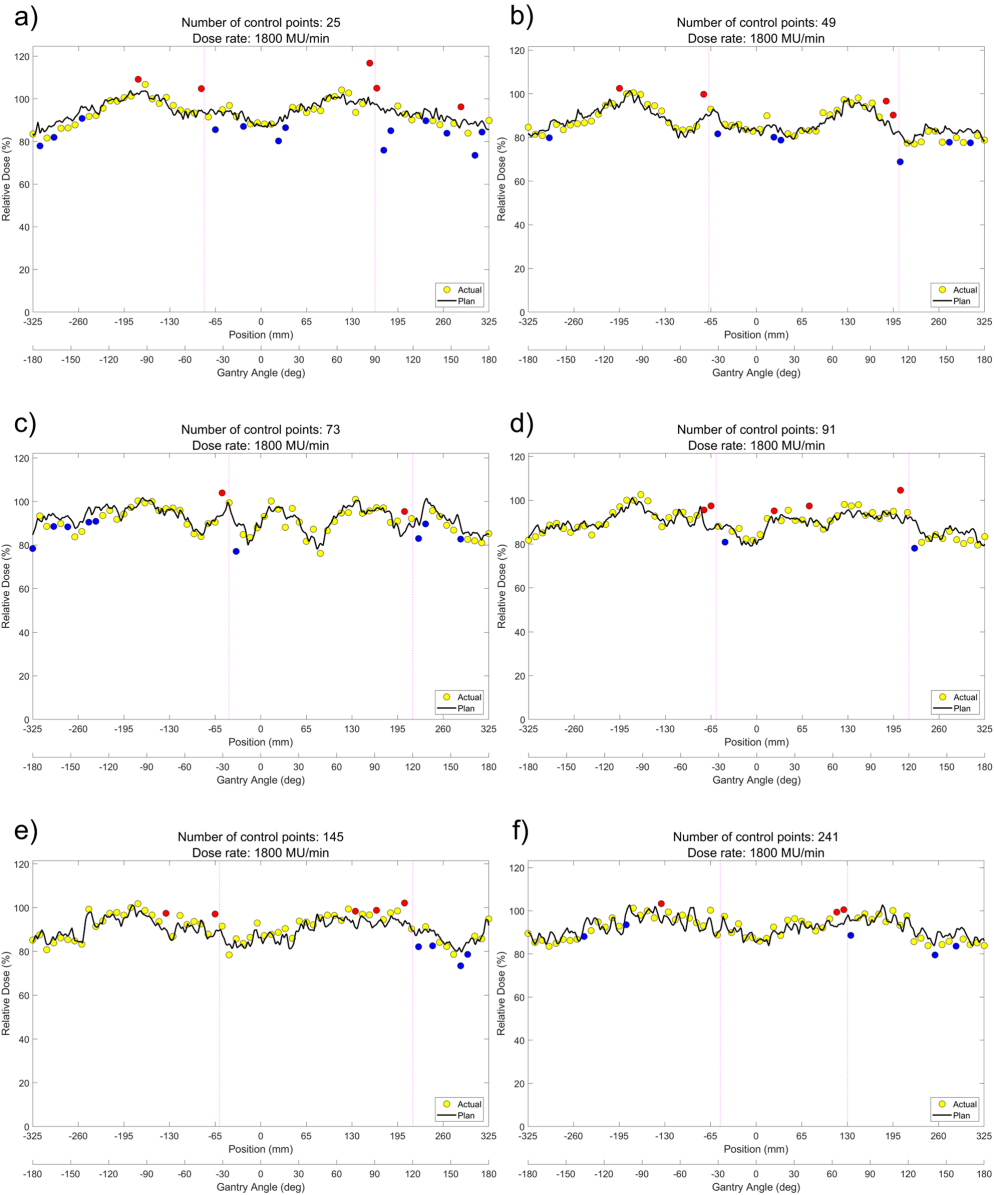
ArcCHECK measurements of 10 MV-FFF DCAT plans with different numbers of control points. (**a**) 25, (**b**) 49, (**c**) 73, (**d**) 91, (**e**) 145, and (**f**) 241.

**Figure 3:**
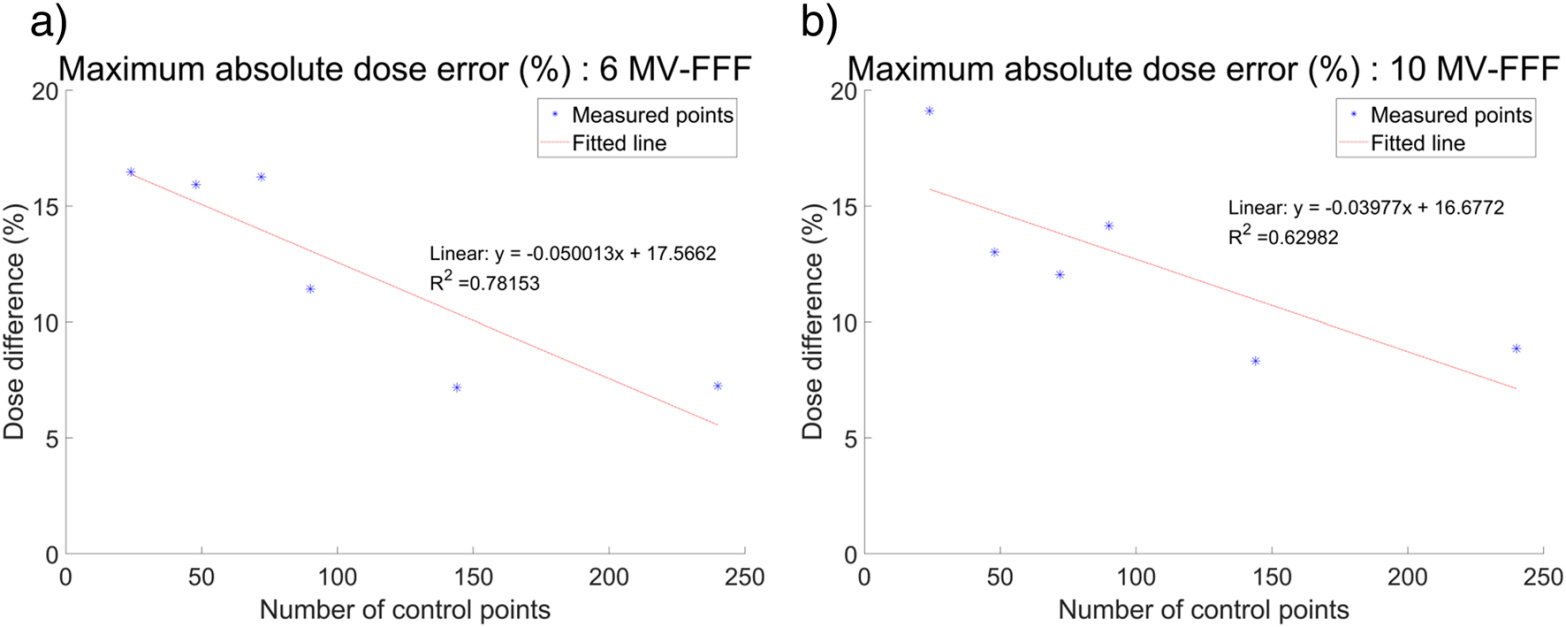
Maximum absolute dose error at inter-segment breakpoints according to the number of control points measured by ArcCHECK, and linear fitted line with R-squared values. (**a**) 6 MV-FFF, (**b**) 10 MV-FFF.

ArcCHECK measurements of DCAT plans according to the different number of control points are shown in Fig. 1a–f (6 MV-FFF) and Fig. 2a–f (10 MV-FFF), respectively. The data points colored yellow represent ArcCHECK measured data, while the black solid lines show the reference data for the comparison, extracted from the TPS 3D dose to a cylindrical dose plane. Hot spots where the measurement was > 5% higher are colored red, and cold spots where the result was > 5% lower were colored blue. The intrinsic inter-segment break for every 1000 MU is indicated by magenta dashed line. Using ArcCHECK measurements, one could observe dose differences due to MU delivery errors at the inter-segment breakpoints, and the DCAT plan with a smaller number of control points showed a larger dose difference for both 6 MV-FFF and 10 MV-FFF. The gamma passing rate (GPR) with a 3%/3 mm local gamma criterion (10% low-dose threshold) for ArcCHECK measurements of DCAT plans according to several control points is summarized in Table 2, and the same tendency was confirmed. In the 6 MV-FFF DCAT plans, the highest GPR was the 98.5% when the number of control points was 241. Conversely, the GPR was 90.8% at 25, the lowest number of control points. Similarly, in the 10 MV-FFF DCAT plans, as the number of control points decreased, the GPR decreased from 94.6 to 84.6%. The maximum absolute error at the inter-segment breakpoint according to the number of control points is plotted in Fig. 3, and the linear fitted line shows that the dose error is inversely proportional to the number of control points.

**Table 2:** GPR of ArcCHECK measurements according to the number of control points.

Number of control points	25	49	73	91	145	241
*GPR (%)*						
6 MV-FFF	90.8	92.4	93.1	93.1	97.0	98.5
10 MV-FFF	84.6	89.2	90.8	91.5	89.2	94.6

**Figure 4:**
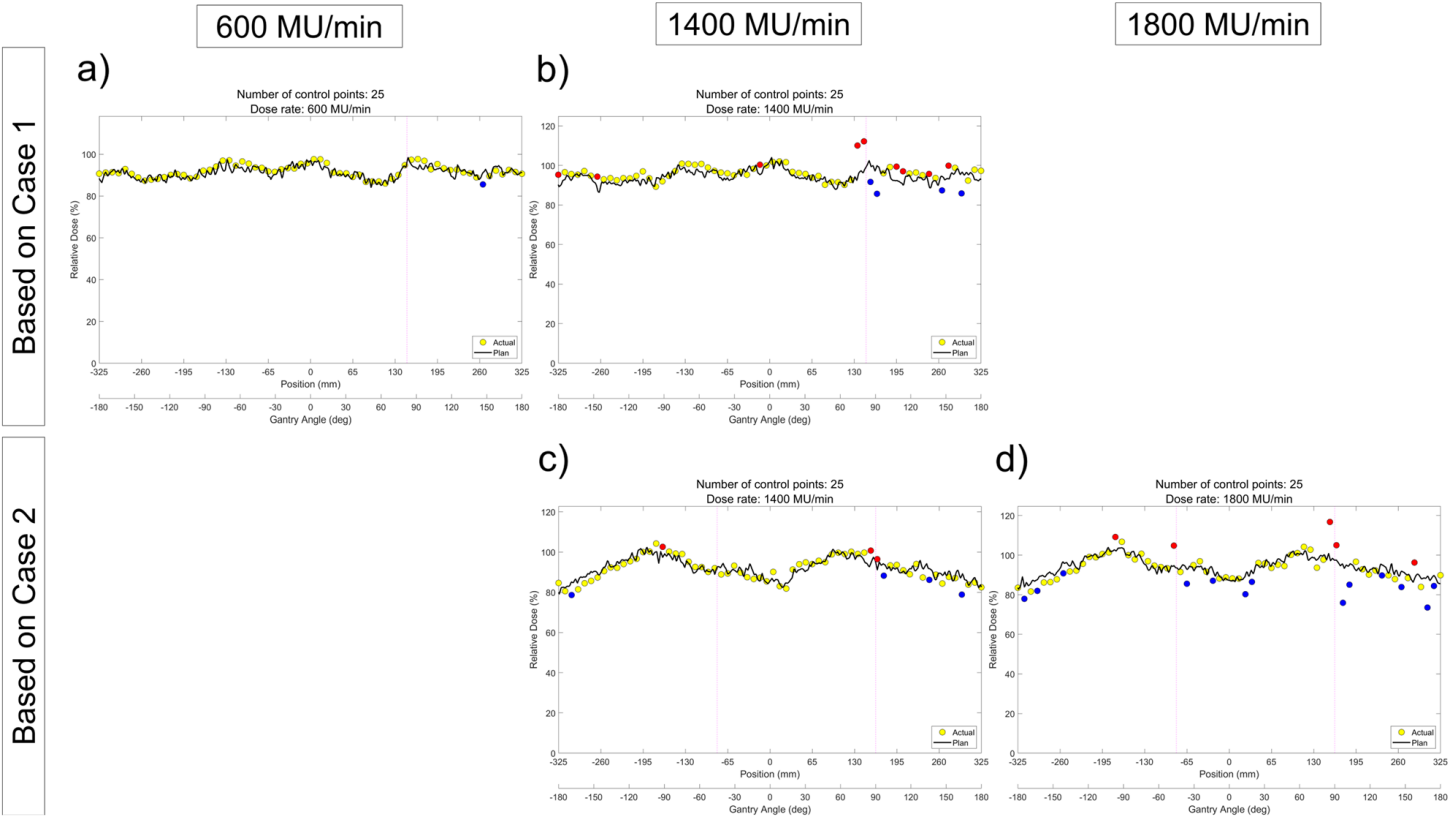
ArcCHECK measurements of DCAT plans with different dose rate. (**a**) 600 MU/min (6 MV), (**b**) 1400 MU/min (6 MV-FFF), (**c**) 1400 MU/min (6 MV-FFF), and (**d**) 1800 MU/min (10 MV-FFF), respectively. (**a**,**b**) are based on the plan of Case 1, and (**c**,**d**) are based on the plan of Case 2. The number of control points for all plans was fixed as 25.

**Figure 5:**
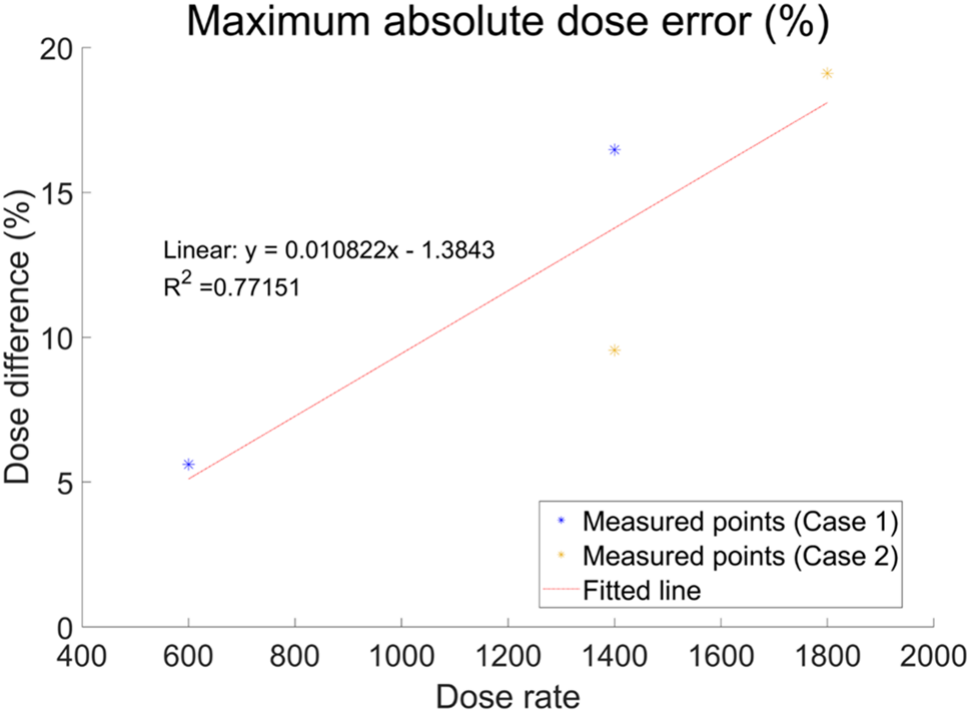
Maximum absolute dose error at inter-segment breakpoints according to the dose rate measured by ArcCHECK, and a linear fitted line with R-squared value.

The ArcCHECK measurements of the DCAT plans according to the dose rate are shown in Fig. 4. As the dose rate increased, we observed that the dose difference at the inter-segment breakpoint became larger, and under this influence, the GPR decreased from 98.5 to 90.8% in Case 1, and from 93.1 to 84.6% in Case 2, respectively, as summarized in Table 3. Furthermore, as shown in Fig. 5, it was also confirmed that the maximum absolute dose error is linearly proportional to the dose rate.

**Table 3:** GPR of ArcCHECK measurements according to the dose rate. The number of control points for all plans was fixed as 25.

Dose rate (MU/min)	600	1400	1800
*GPR (%)*			
Based on Case 1	98.5	90.8	
Base on Case 2		93.1	84.6

For further investigation as mentioned in the Methods section, the deliverability of DCAT according to the plan parameters were also evaluated using the treatment machine log file. The similar tendency consistent with the ArcCHECK results were observed, which further support the contents of this study. Detailed results are provided in Supplementary Material B.

### Evaluation of dosimetric impact according to the deliverability

**Figure 6:**
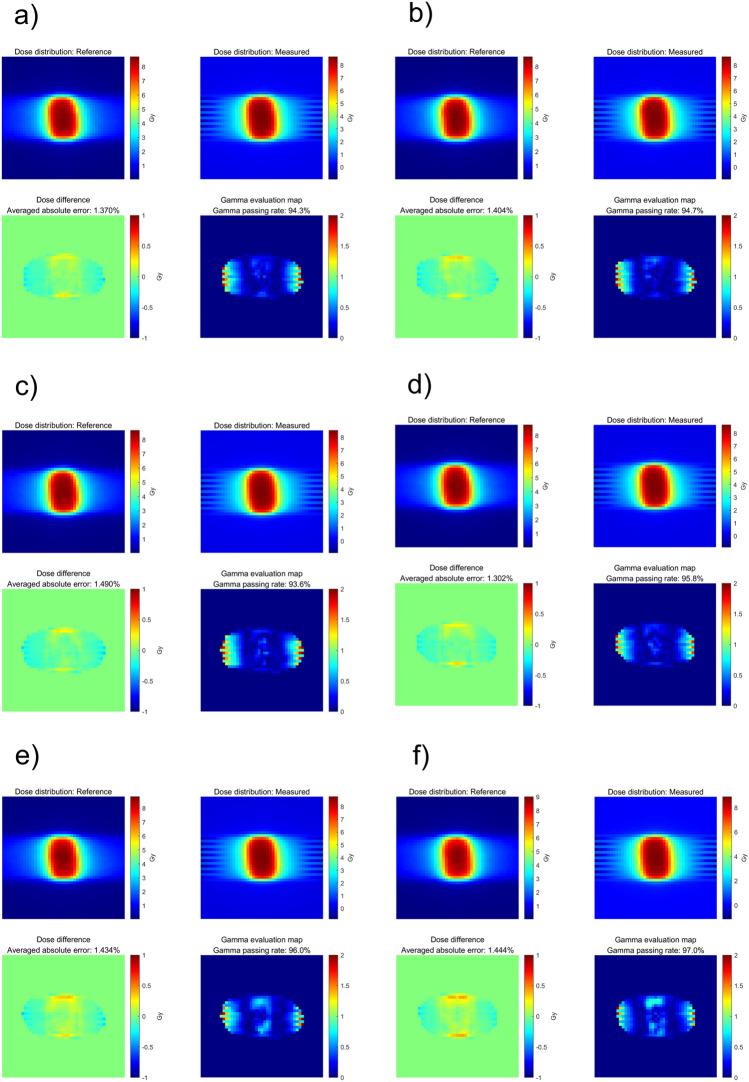
Two-dimensional distributions of computed dose and measured dose by Octavius 1000 SRS and gamma indices for Case 1 (6 MV-FFF), according to different numbers of control points (3%/3 mm local gamma criterion (10% low-dose threshold)). (**a**) 25, (**b**) 49, (**c**) 73, (**d**) 91, (**e**) 145, and (**f**) 241.

**Figure 7:**
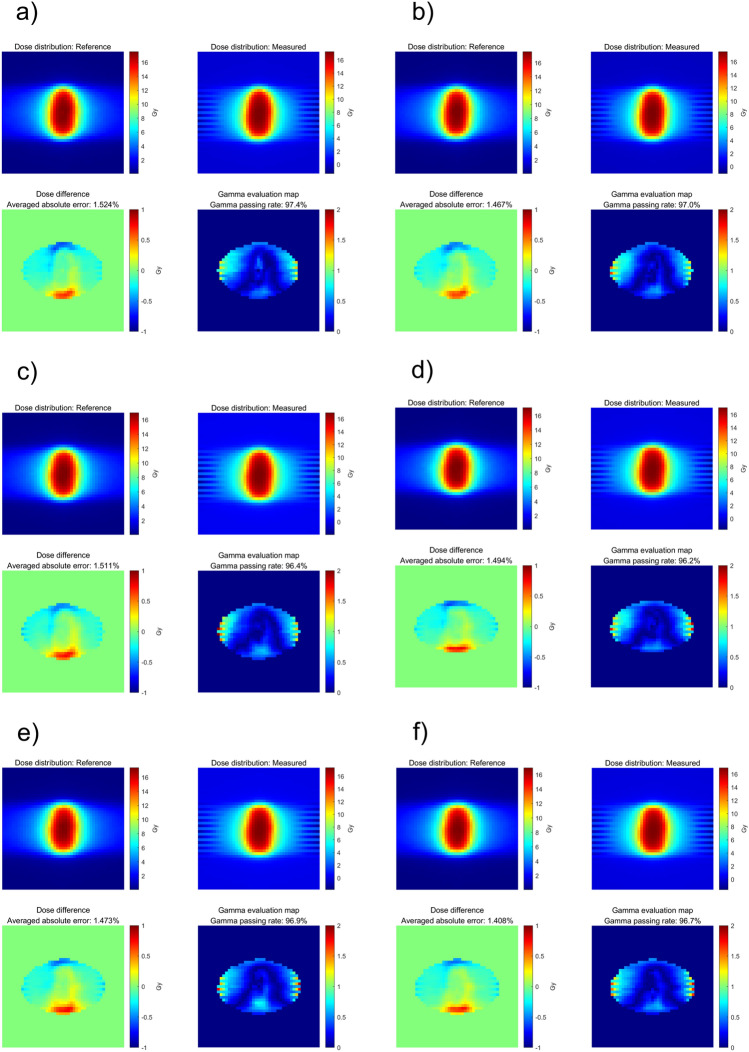
Two-dimensional distributions of computed dose and measured dose by Octavius 1000 SRS and gamma indices for Case 2 (10 MV-FFF), according to different numbers of control points (3%/3 mm local gamma criterion (10% low-dose threshold)). (**a**) 25, (**b**) 49, (**c**) 73, (**d**) 91, (**e**) 145, and (**f**) 241.

**Figure 8:**
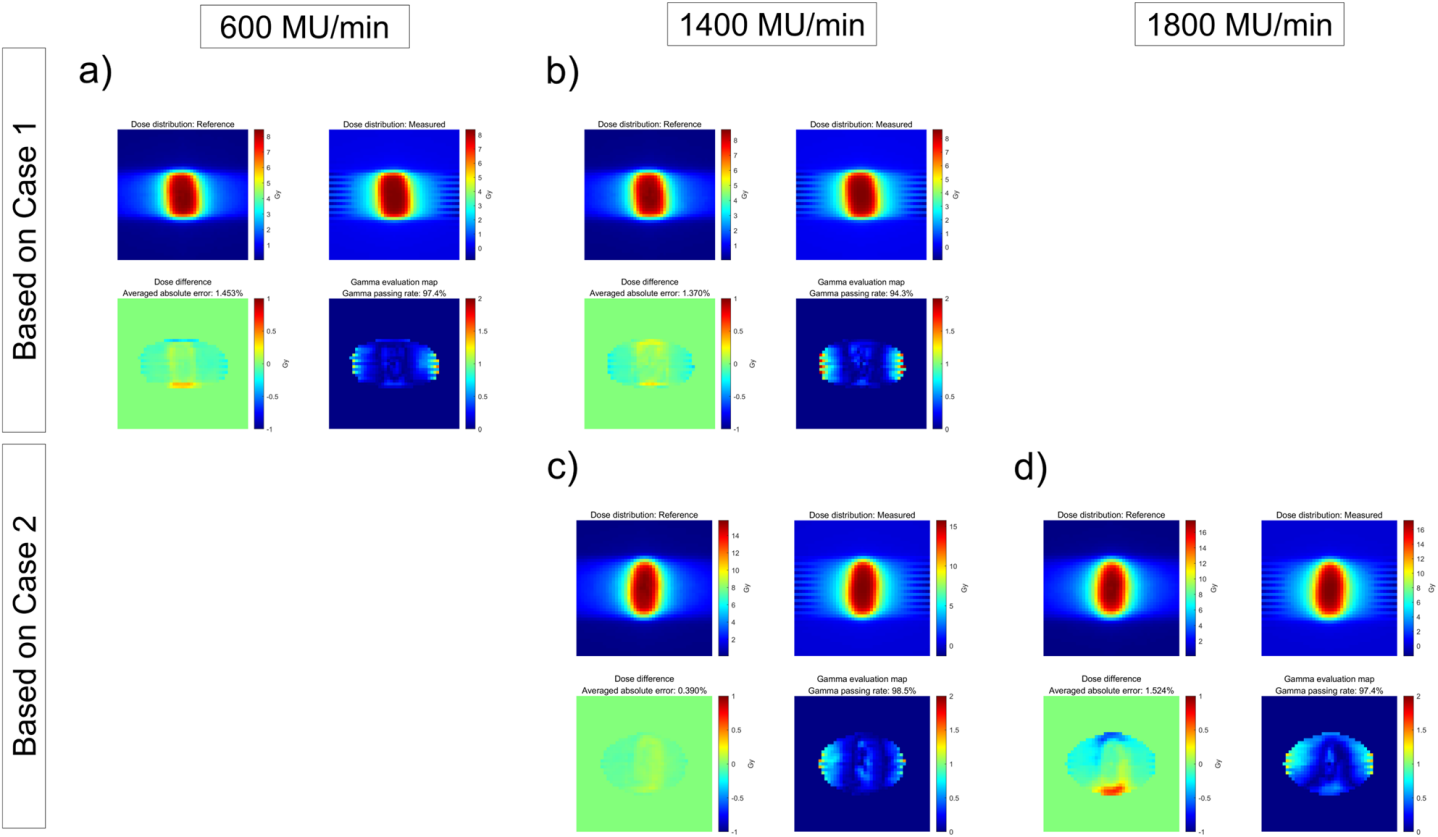
Two-dimensional distributions of computed dose and measured dose by Octavius 1000 SRS and gamma indices according to different dose rates (a) 600 MU/min (6 MV), (b) 1,400 MU/min (6 MV-FFF), (**c**) 1400 MU/min (6 MV-FFF), and (**d**) 1800 MU/min (10 MV-FFF), respectively. (**a**,**b**) are based on the plan of Case 1; (**c**,**d**) are from Case 2. (3%/3 mm local gamma criterion (10% low-dose threshold)).

To determine the dosimetric influence of the number of control points and dose rate in the DCAT plan, dose measurements were performed using the Octavius 1000 SRS. The gamma analysis results between the computed and measured doses are summarized in Table 4 and 5, respectively. Similar to ‘Deliverability of DCAT according to the plan parameters’ in the Results section, the GPR varies depending on the planning parameters. Specifically, a high number of control points and a low dose rate resulted in a high GPR. This was also confirmed in the two-dimensional distribution of computed and measured dose and gamma evaluation maps, as shown in Figs. 6, 7, and 8 (3%/3 mm local gamma criterion (10% low-dose threshold)) and Supplemental Figures S.C.1–S.C.3 (1%/1 mm local gamma criterion (10% low-dose threshold)). For the detailed analysis with 1%/1 mm gamma evaluation, it is summarized in Supplementary Material C. Although the degree of agreement between the computed and measured doses differed between the two cases, a similar trend was observed.

**Table 4:** GPRs of Octavius 1000 SRS results according to the number of control points.

Number of control points		25	49	73	91	145	241
*GPR (%)*							
6 MV-FFF	3%/3 mm	94.3	94.7	93.6	95.8	96.0	97.0
	1%/1 mm	85.5	84.8	80.9	88.2	88.1	89.6
10 MV-FFF	3%/3 mm	97.4	97.0	96.4	96.2	96.9	96.7
	1%/1 mm	87.2	85.5	86.8	85.4	90.0	90.7

**Table 5:** GPRs of Octavius 1000 SRS results according to dose rate.

	Dose rate (MU/min)	600	1400	1800
*GPR (%)*				
Based on Case 1	3%/3 mm	100.0	98.5	
	1%/1 mm	99.9	89.4	
Base on Case 2	3%/3 mm		100.0	99.5
	1%/1 mm		95.4	87.7

Dose simulations in the patient domain by using Mobius3D with a modified RT-plan showed a similar impact on deteriorated deliverability. Likewise, we observed that the GPR varied depending on the planning parameters. A lower number of control points and a higher dose rate resulted in a low GPR. Regarding the dose impact by specific region, for targets located primarily in the isocenter, the dose differences were negligible, whereas for organs at risk (OAR) located mainly off-isocenter, the dose differences were significant up to − 8.77%. Detailed analyses are described in Supplementary Material D.

## Discussions

We investigated the impact of gantry wobble caused by the intrinsic inter-segment breaks of the Elekta LINAC on the deliverability of the DCAT plan and the resultant dose delivered to the patient. The deliverability of the DCAT plan according to the number of control points and dose rate was evaluated. As expected, it was verified that the plan was adversely affected by gantry wobble, which is parameter-dependent. Furthermore, dose changes according to deteriorated deliverability were found, and it was confirmed that a non-negligible decrease in deliverability and consequent delivery errors could occur below a certain number of control points or above a certain dose rate. The overall results show that radiation treatment based on the DCAT technique in conjunction with Elekta LINAC may deteriorate plan deliverability under certain conditions.

The limitation of this study is that we were unable to investigate the various conditions of dose rate, thus we envision to conduct further studies if available in the future. In the non-clinical conditions, it was feasible to adjust the dose rate to the desired values for artificial plans like squared beam, primarily used for periodic QA and maintenance. However, it was challenging to individually adjust the dose rate in TPS and clinical treatment console. Consequently, in this study, we simply adjusted the X-ray energy to change the dose rate, enabling the application of different maximum dose rates without compromising the optimized plans and their quality.

In this paper, as the study was conducted with different plan parameter setting based on only two patient cases, the small number of cases is a weakness of the study. Therefore, the goal of future research will include to increase the number of cases with a variety of diseases. However, considering the appropriate cases in which DCAT plan is clinically applicable, we believe that the results of the current study are sufficient to evaluate the tendency of deliverability according to plan parameters in the clinical circumstances at out institute at this moment.

This inherent limitation should be fully acknowledged in advance by a responsible professional such as a medical physicist, and these characteristics should be considered during treatment planning. In other words, it is necessary to select an appropriate number of control points and dose rates to minimize MU delivery errors. However, even for LINACs from the same manufacturer, the variation in DCAT deliverability according to the plan parameters may vary depending on the age and mechanical maintenance of the LINAC. Here, the DCAT deliverability was evaluated on the Elekta Versa HD, which has been installed and in use since 2019. Based on the experience at our institute, it is appropriate to employ more than 73 control points for 6 MV-FFF and more than 91 points for 10 MV-FFF to reduce delivery errors due to gantry wobble to a tolerable level. Related to the dose rate, it is recommended in terms of deliverability to avoid the maximum dose rate that can be used for the corresponding energy.

Therefore, the deliverability of the DCAT plan and the resulting dose errors at the patient-specific pretreatment QA step should be confirmed. However, conventional QA methods, such as two-dimensional array- or chamber-based methods that measure two-dimensional dose distributions or point doses, can only obtain a dose integrated at all gantry angles. Thus, with conventional QA methods, it may be difficult to precisely identify deteriorated deliverability and the resulting dose error. Therefore, the use of semi-3D dosimetry devices such as ArcCHECK or machine log file-based methods that allow for gantry-specific measurement and analysis is recommended.

To evaluate the dosimetric impact in the patient domain, Mobius3D, a dose-volume-histogram (DVH)-based patient-specific QA software, was utilized. The MU weights of the RT-plan were revised according to the measured delivery error for each plan parameter, and the dose was recalculated using the modified RT-plan. The dosimetric effects of the delivery error for each organ structure are summarized in Supplementary Material D. The dose variations of the target were all within 0.3% in this study. This means that, even if MU delivery errors due to gantry wobble occur, the resultant dose impact would be averaged and become negligible because of the characteristics of arc therapy, which delivers the dose to the isocenter evenly at all gantry angles. In contrast, normal organs are usually placed off-center, unlike targets that are mostly located in the isocenters. Thus, depending on where the gantry wobble occurs, dose differences in normal organs can occur, which in this study were usually approximately a few percent (up to 10%). In terms of the percentage, the value was not small; however, when assessed from an absolute dose standpoint, it may have remained relatively modest. Therefore, at this stage, the direct association between the clinical impact of the dose-delivery errors identified in this study may be limited.

## Conclusions

We demonstrated that the deliverability of the DCAT plan and the resultant dose delivered to the patient are affected by inter-segment breaks and the intrinsic characteristics of the Elekta LINAC. Therefore, when performing radiation treatment on the Elekta LINAC using the DCAT technique, appropriate values of plan parameters, such as the number of control points and dose rate that affect the deliverability of DCAT, should be determined, and gantry angle-specific measurements and verifications should be performed before the treatment.

### Supplementary Information

Supplementary Information.

## Data Availability

The datasets used and/or analyzed during the current study are available from the corresponding author on reasonable request.
