# Supplementary material for: Evaluation of the deliverability of dynamic conformal arc therapy (DCAT) by gantry wobble and its influence on dose
Source: Sci Rep. 2024 Mar 26;14:7134. doi: 10.1038/s41598-024-57644-4 (PMC10965989; doi:10.1038/s41598-024-57644-4)
Supplement: Supplementary file 1 — Supplementary Information. [file 41598_2024_57644_MOESM1_ESM.docx]

# Supplemental Material

**A: Dose distribution of the representative plans in this study**

**
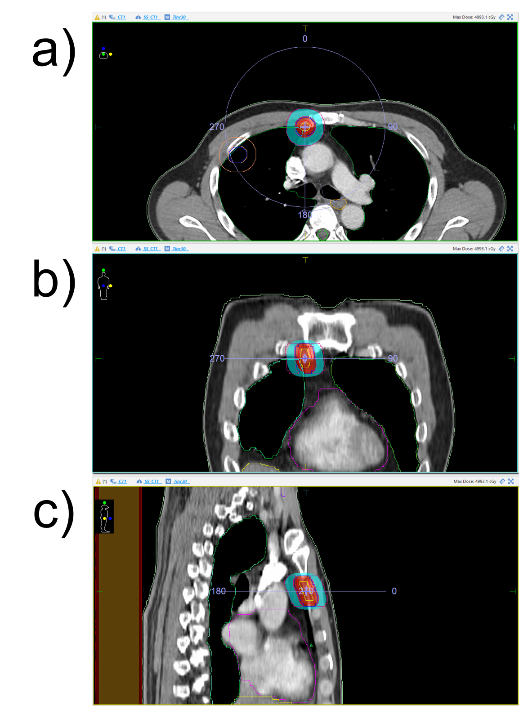
**

**Figure S.A.1.** Dose distribution of (a) axial, (b) coronal, and (c) sagittal view for the representative DCAT plan with 6 MV-FFF utilized in this study. The number of control points was set to 25.

**
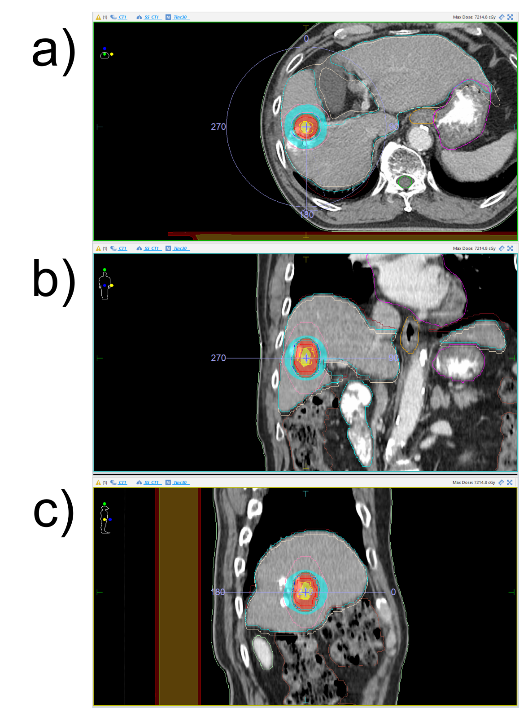
**

**Figure S.A.2.** Dose distribution of (a) axial, (b) coronal, and (c) sagittal view for the representative DCAT plan with 10 MV-FFF utilized in this study. The number of control points was set to 25.

**B: Evaluation of the deliverability of DCAT by using treatment machine log files**

In addition to the measurement-based investigation, the treatment machine log files were analyzed in parallel to evaluate the deliverability of DCAT according to the number of control points and the dose rate. The machine log files were generated using a commercial software called Mobius Log (Varian Medical Systems, Palo Alto, CA), which extracts machine parameters from the LINAC via iCom communication. Among the various machine parameters acquired using this method, the gantry angle and delivered MU per each control point were checked in detail, and the amount of delivery error under different conditions were examined.

The results for different numbers of control points are shown in Figures S.B.1 and S.B.2, and the data according to the dose rate are shown in Figure S.B.3. Delivered MU within each beam segment (between two adjacent control points) were compared with the reference MU of the plan. Consistent with the ArcCHECK measurements, deteriorated deliverability of DCAT and the resultant MU delivery errors were observed in the machine log file at inter-segment breakpoints (highlighted by the dashed circle), with the magnitude of the errors being larger for fewer control points and higher dose rates.


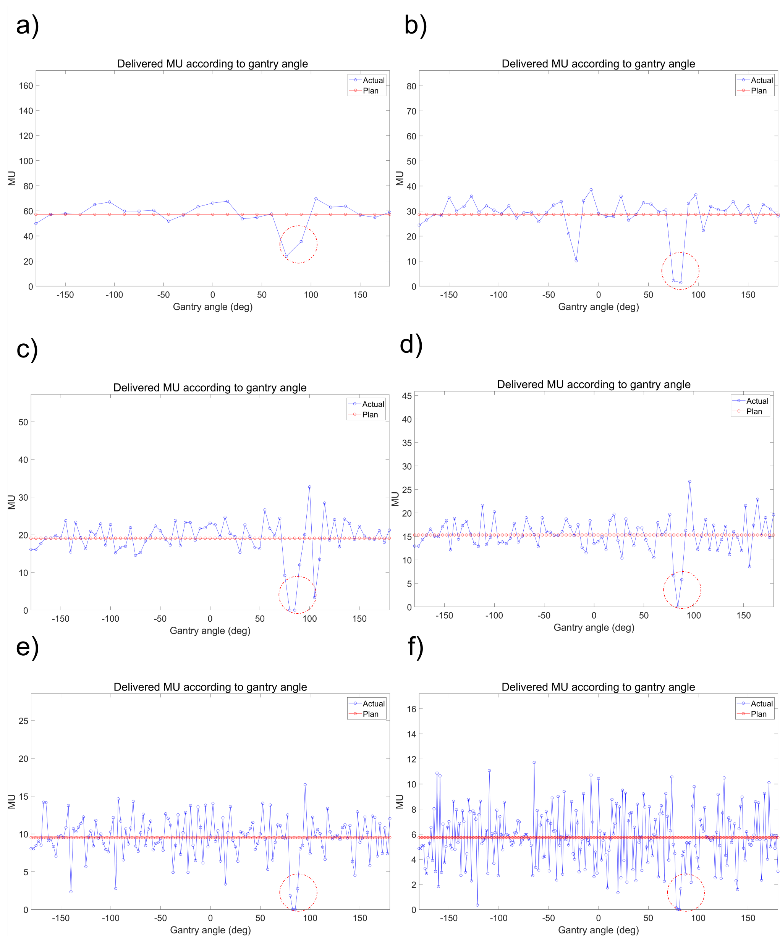


**Figure S.B.1.** Delivered MU according to gantry angle acquired from machine log files of 6 MV-FFF DCAT plans with different numbers of control points. (a) 25, (b) 49, (c) 73, (d) 91, (e) 145, and (f) 241.


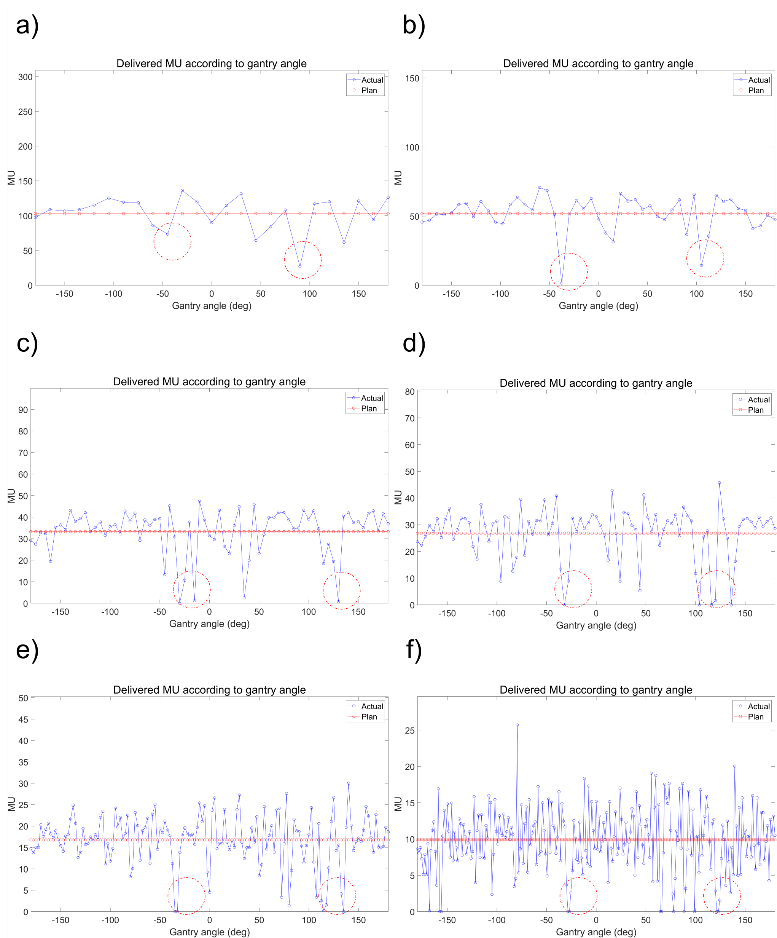


**Figure S.B.2.** Delivered MU according to gantry angle acquired from machine log files of 10 MV-FFF DCAT plans with different numbers of control points. (a) 25, (b) 49, (c) 73, (d) 91, (e) 145, and (f) 241.


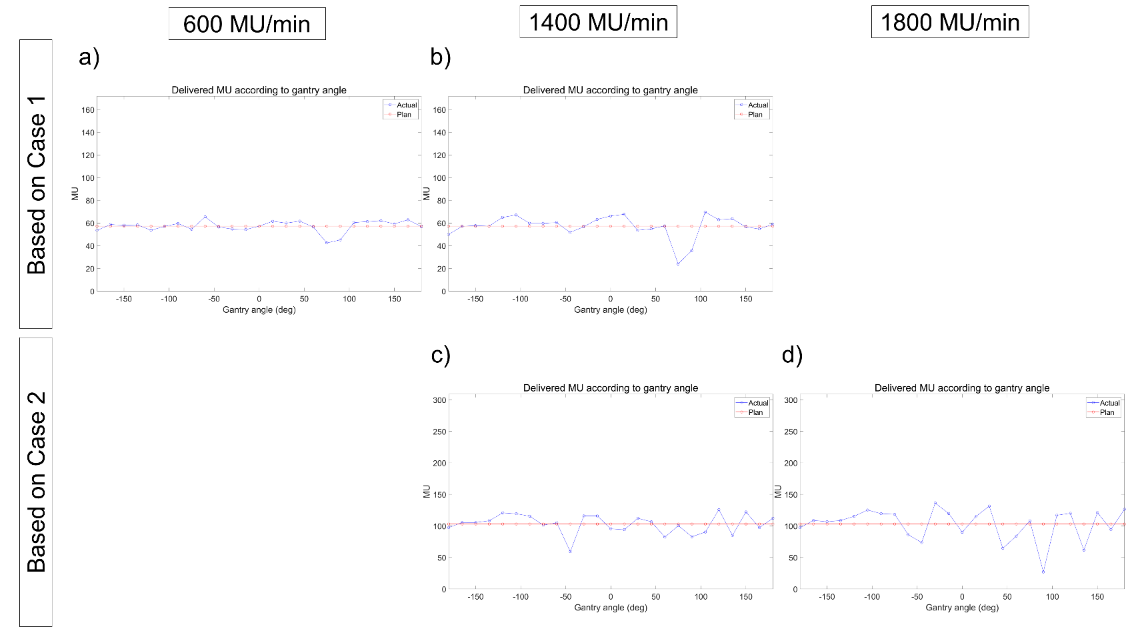


**Figure S.B.3.** Delivered MU according to gantry angle acquired from machine log files of DCAT plans with different dose rates. (a) 6 MV with 600 MU/min, (b) 6 MV-FFF with 1,400 MU/min, (c) 6 MV-FFF with 1,400 MU/min, and (d) 10 MV-FFF with 1,800 MU/min, respectively. (a) and (b) are based on the plan of Case 1; (c) and (d) are from Case 2.

**C: Dosimetric evaluation according to deliverability using Octavius 1000 SRS**

The results of ‘Dose evaluation according to the deliverability’ in the Results section for 1%/1 mm are as below.

**
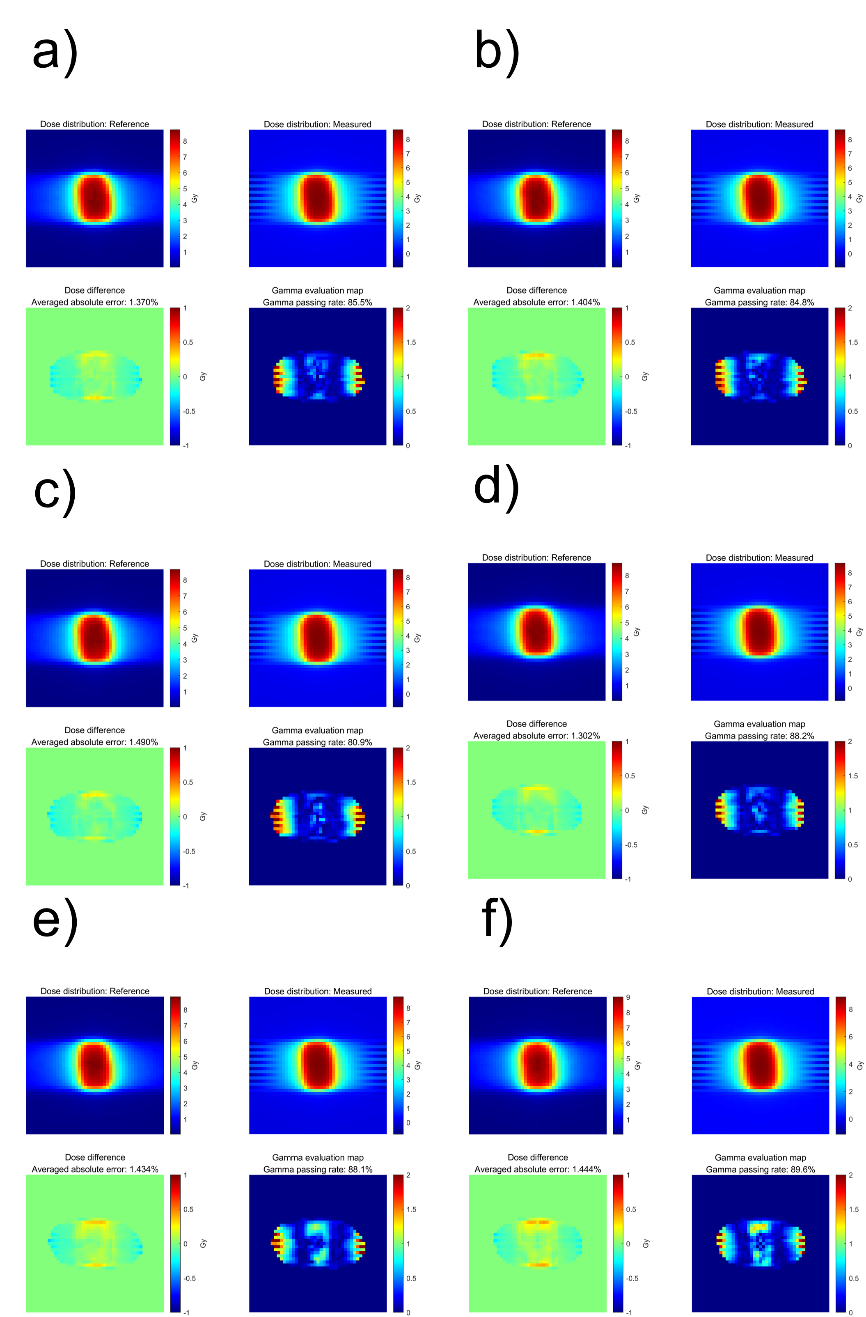
**

**Figure S.C.1.** Two-dimensional distributions of computed dose and measured dose by Octavius 1000 SRS and gamma indices for Case 1 (6 MV-FFF), according to different numbers of control points (1%/1 mm gamma criterion (10% low-dose threshold)). (a) 25, (b) 49, (c) 73, (d) 91, (e) 145, and (f) 241.

**
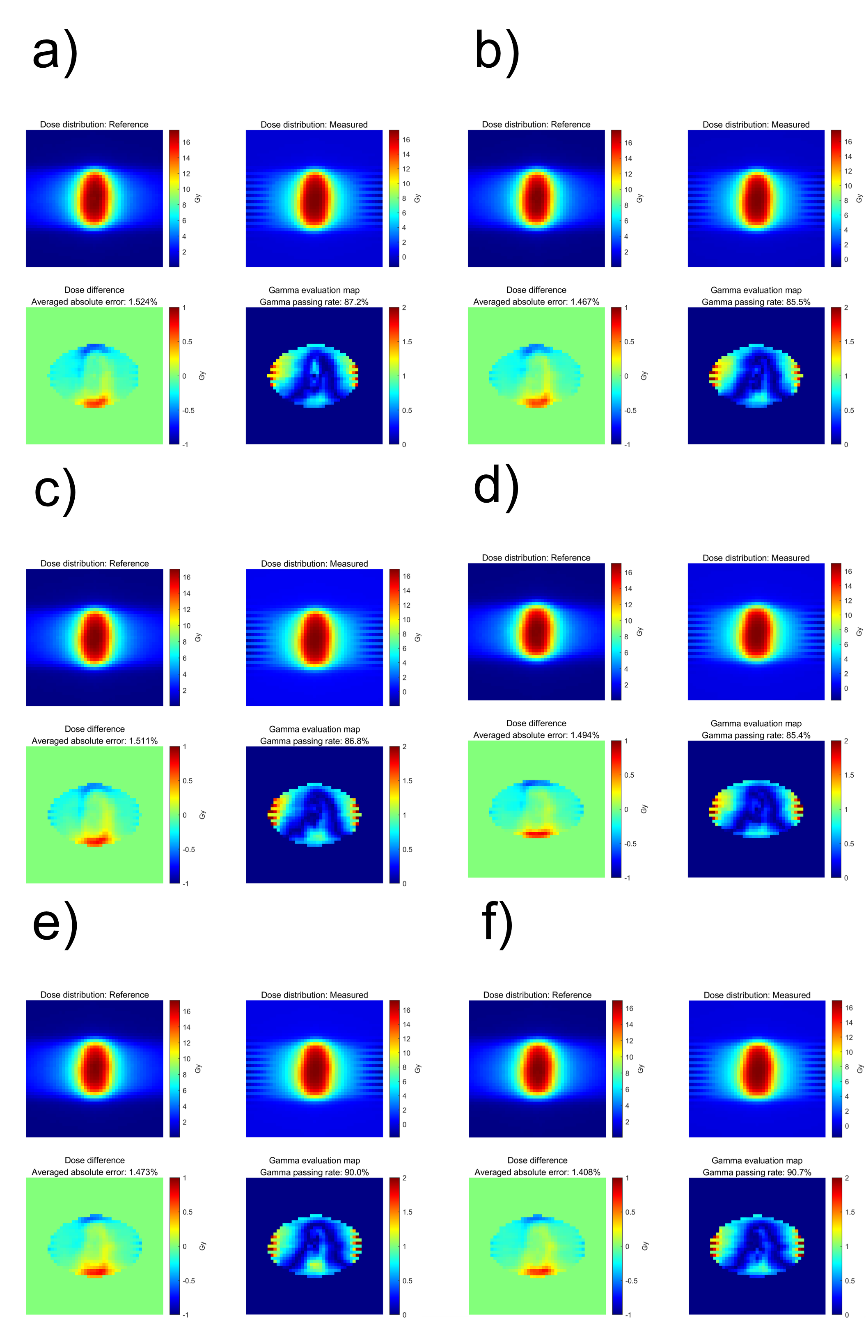
**

**Figure S.C.2.** Two-dimensional distributions of computed dose and measured dose by Octavius 1000 SRS and gamma indices for Case 2 (10 MV-FFF), according to different numbers of control points (1%/1 mm gamma criterion (10% low-dose threshold)). (a) 25, (b) 49, (c) 73, (d) 91, (e) 145, and (f) 241.


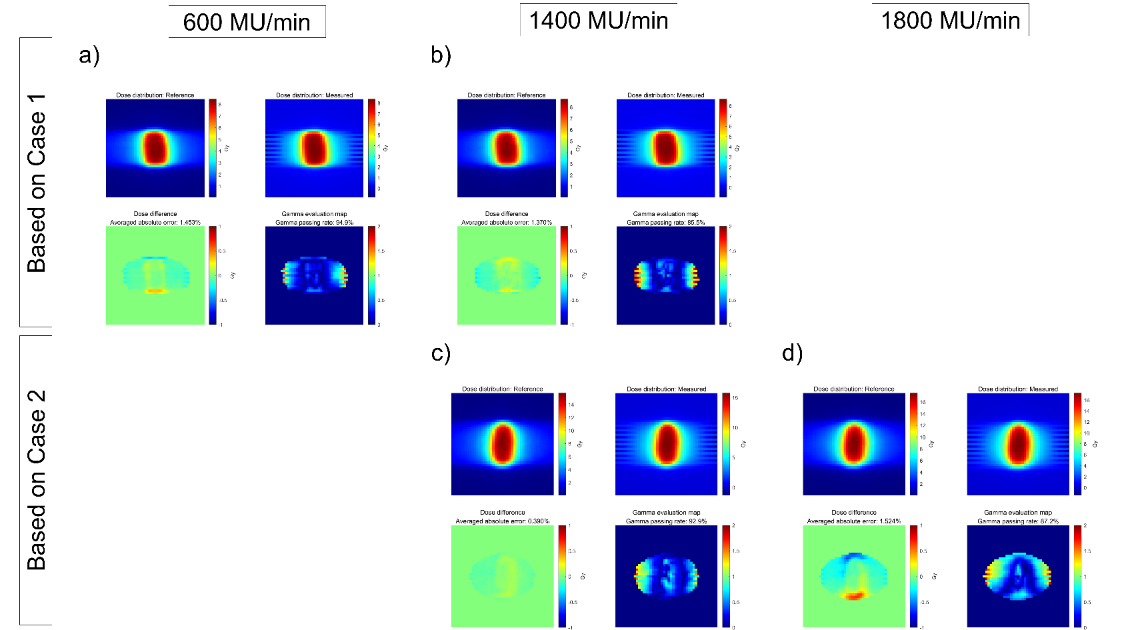


**Figure S.C.3.** Two-dimensional distributions of computed dose and measured dose by Octavius 1000 SRS and gamma indices according to different dose rates (a) 6 MV with 600 MU/min, (b) 6 MV-FFF with 1,400 MU/min, (c) 6 MV-FFF with 1,400 MU/min, and (d) 10 MV-FFF with 1,800 MU/min, respectively. (a) and (b) are based on the plan of Case 1; (c) and (d) from Case 2. (1%/1 mm gamma criterion (10% low-dose threshold))

**D: Dosimetric evaluation according to deliverability using a modified RT-plan with Mobius3D**

The effect of deteriorated deliverability on patient dose was simulated through dose re-calculation with a modified RT-plan that changed the monitor unit (MU) weights according to the measured delivery errors. As described in the Method section, modified RT-plans were generated by incorporating machine log files for each parameter condition into the ‘Cumulative meterset weight’ for each control point, instead of originally planned values, as shown in Figure S.D.1. Since the TPS used in clinical practice is restricted from importing externally modified RT-plans for safety reasons, this study utilized dose calculation engine of Mobius3D instead to determine the dose impact of delivery errors in the patient domain.

We performed a gamma analysis of three-dimensional doses calculated between the original and modified plans with simulated delivery errors, as shown in Figure S.D.2-S.D.4 (3%/3 mm gamma criterion) and Supplemental Figures S.D.5-S.D.7 (1%/1 mm gamma criterion). The gamma analysis results are summarized in Table S.D.1 and S.D.2, respectively. As in the analysis based on the Octavius measurement data, we observed that the GPR varied depending on the planning parameters. A lower number of control points and a higher dose rate resulted in a low GPR.

|  | GPR (%) | | | | | | |
| --- | --- | --- | --- | --- | --- | --- | --- |
|  | Number of control points | 25 | 49 | 73 | 91 | 145 | 241 |
| 6 MV-FFF | 3%/3 mm | 98.5 | 99.4 | 99.8 | 99.8 | 100.0 | 100.0 |
|  | 1%/1 mm | 89.4 | 95.1 | 97.8 | 98.2 | 99.7 | 100.0 |
| 10 MV-FFF | 3%/3 mm | 99.5 | 100.0 | 100.0 | 100.0 | 100.0 | 100.0 |
|  | 1%/1 mm | 87.7 | 95.5 | 96.6 | 98.8 | 99.6 | 100.0 |

**Table S.D.1.** GPRs of Mobius3D results of the modified plan with simulated delivery errors according to the number of control points.

| GPR (%) | | | | |
| --- | --- | --- | --- | --- |
|  | Dose rate  (MU/min | 600 | 1400 | 1800 |
| Based on Case 1 | 3%/3 mm | 100.0 | 98.5 |  |
|  | 1%/1 mm | 99.9 | 89.4 |  |
| Base on Case 2 | 3%/3 mm |  | 100.0 | 99.5 |
|  | 1%/1 mm |  | 95.4 | 87.7 |

**Table S.D.2.** GPRs of Mobius3D results of the modified plan with simulated delivery errors according to dose rate.


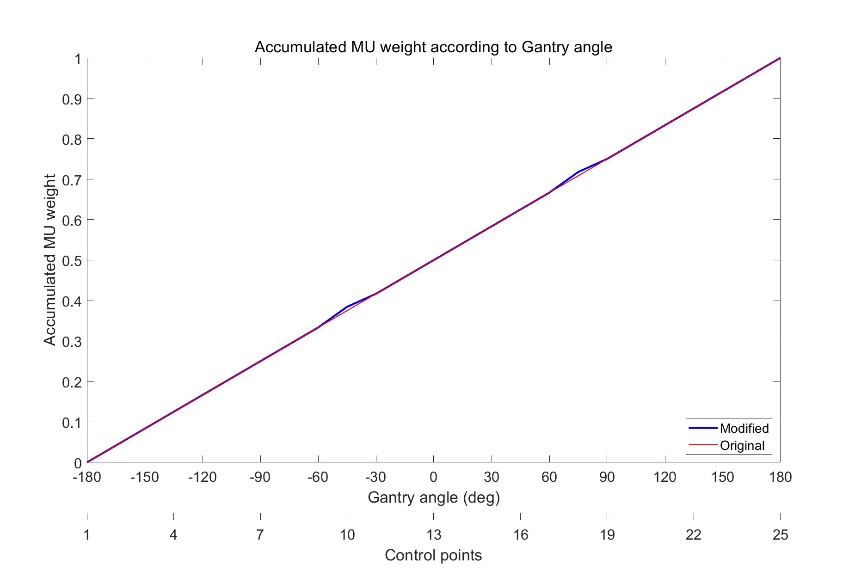


**Figure S.D.1.** The example of cumulative meterset weight of the original and modified plan with simulated delivery errors. This example pertains to Case 2 (10 MV-FFF) with the number of control points as 25.


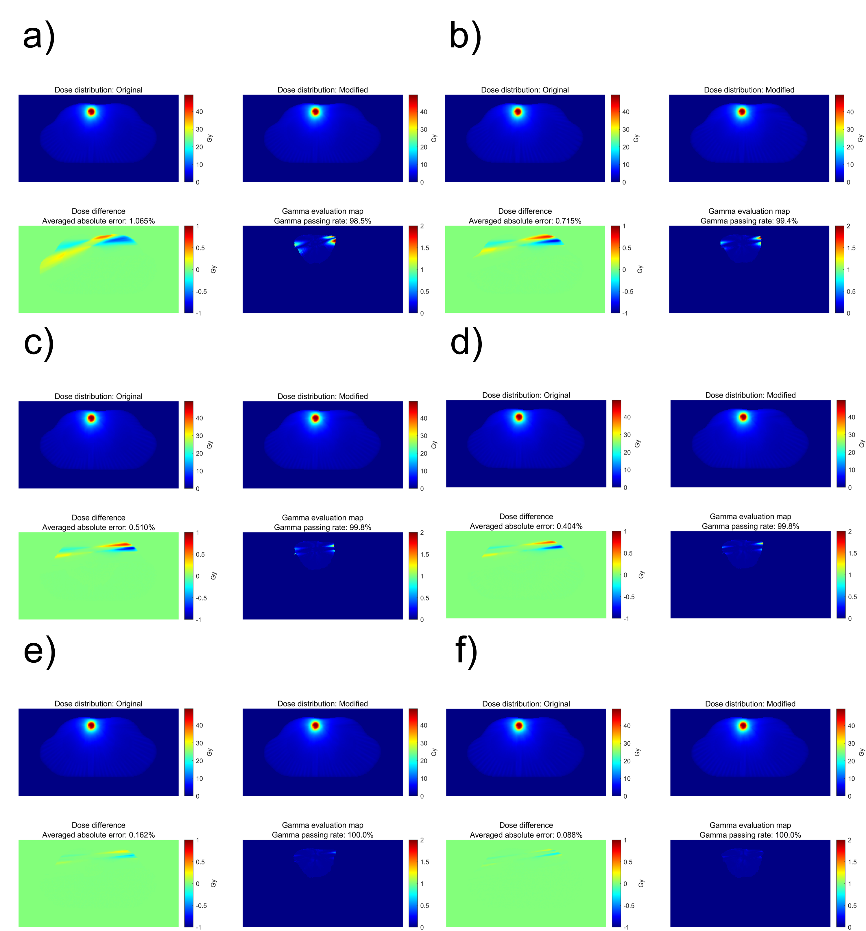


**Figure S.D.2.** Two-dimensional distributions of the modified plan with simulated delivery errors and gamma indices for Case 1 (6 MV-FFF), according to different numbers of control points (3%/3 mm gamma criterion (10% low-dose threshold)). (a) 25, (b) 49, (c) 73, (d) 91, (e) 145, and (f) 241.


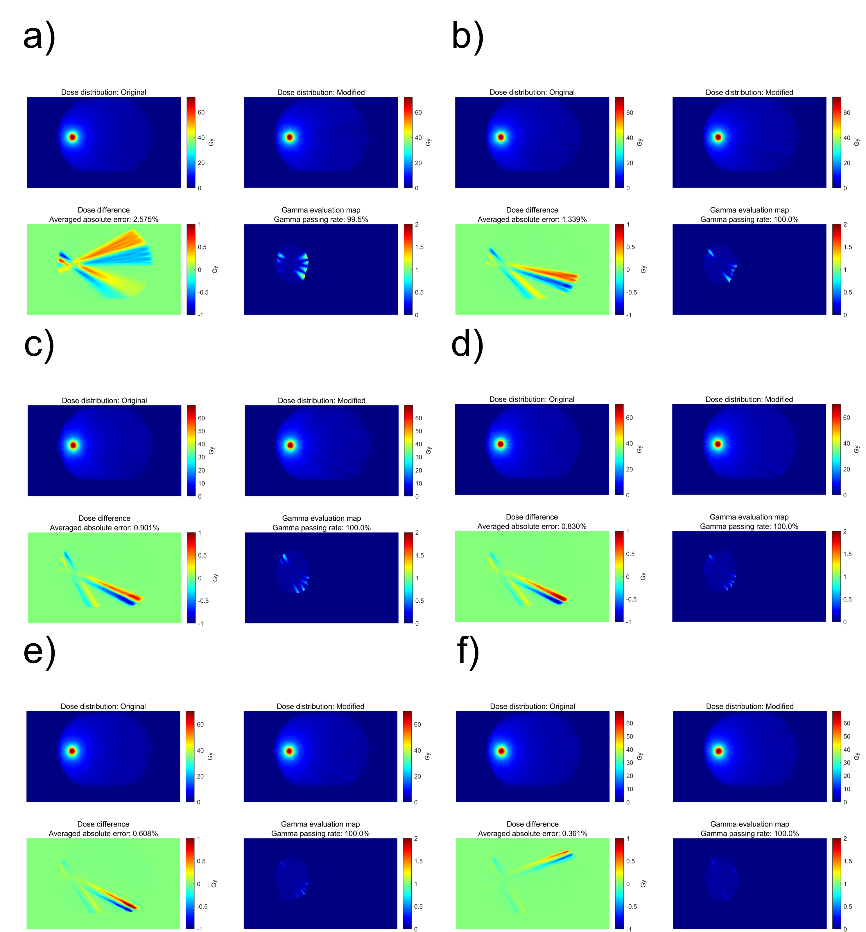


**Figure S.D.3.** Two-dimensional distributions of the modified plan with simulated delivery errors and gamma indices for Case 2 (10 MV-FFF), according to different numbers of control points (3%/3 mm gamma criterion (10% low-dose threshold)). (a) 25, (b) 49, (c) 73, (d) 91, (e) 145, and (f) 241.


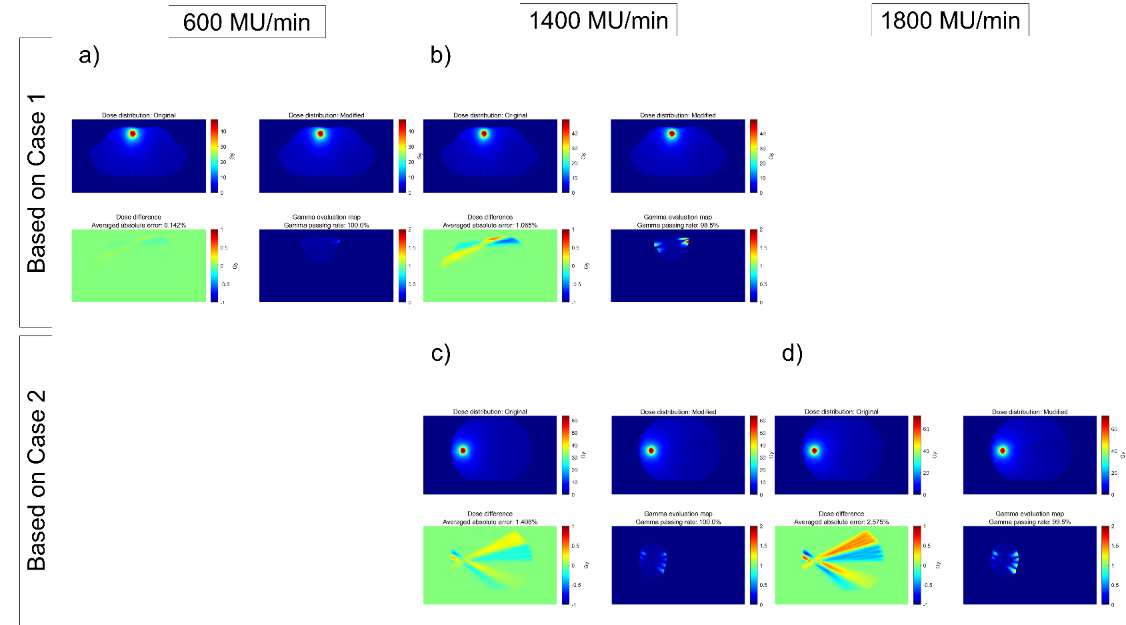


**Figure S.D.4.** Two-dimensional distributions of the modified plan with simulated delivery errors and gamma indices according to different dose rate (a) 6 MV with 600 MU/min, (b) 6 MV-FFF with 1,400 MU/min, (c) 6 MV-FFF with 1,400 MU/min, and (d) 10 MV-FFF with 1,800 MU/min, respectively. (a) and (b) are based on the plan of Case 1; (c) and (d) are from Case 2. (3%/3 mm gamma criterion (10% low-dose threshold))

**
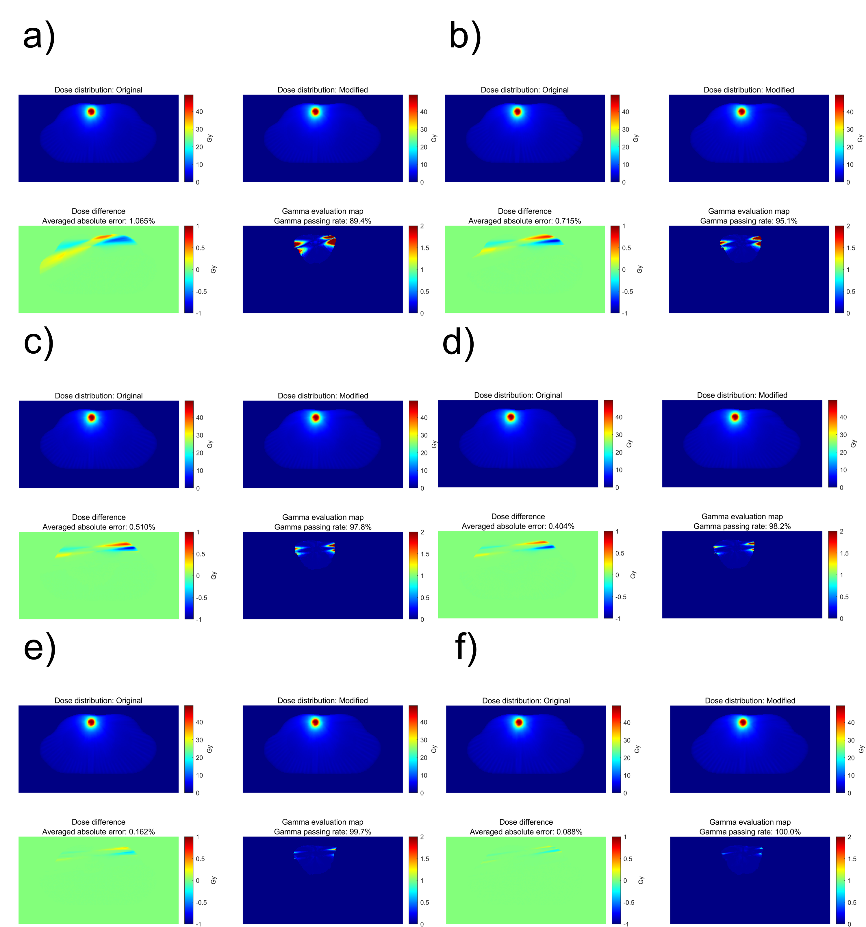
**

**Figure S.D.5.** Two-dimensional distributions of the modified plan with simulated delivery errors and gamma indices for Case 1 (6 MV-FFF), according to different numbers of control points (1%/1 mm gamma criterion (10% low-dose threshold)). (a) 25, (b) 49, (c) 73, (d) 91, (e) 145, and (f) 241.

**
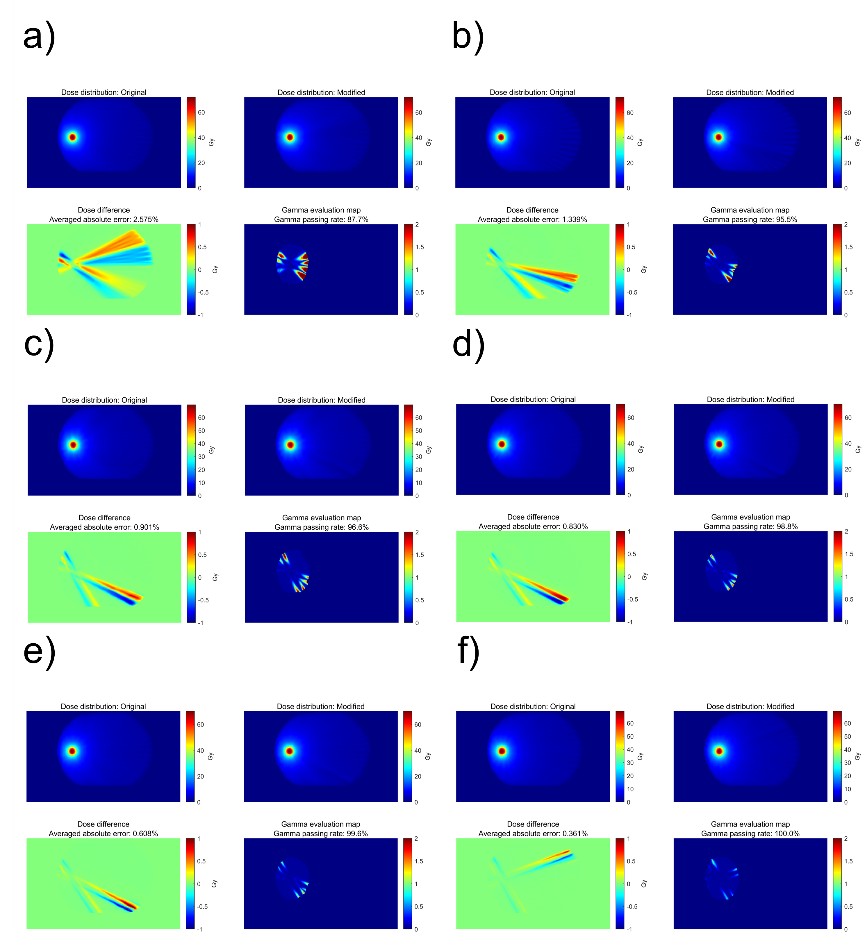
**

**Figure S.D.6.** Two-dimensional distributions of the modified plan with simulated delivery errors and gamma indices for Case 2 (10 MV-FFF), according to different numbers of control points (1%/1 mm gamma criterion (10% low-dose threshold)). (a) 25, (b) 49, (c) 73, (d) 91, (e) 145, and (f) 241.

**
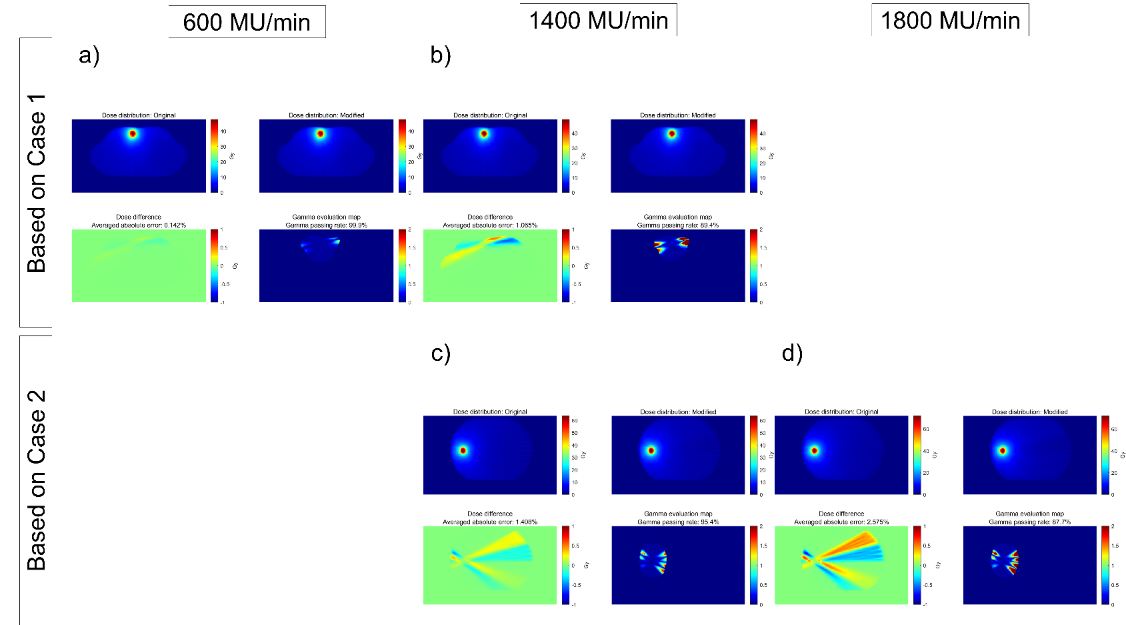
**

**Figure S.D.7.** Two-dimensional distributions of the modified plan with simulated delivery errors and gamma indices according to different dose rates (a) 6 MV with 600 MU/min, (b) 6 MV-FFF with 1,400 MU/min, (c) 6 MV-FFF with 1,400 MU/min, and (d) 10 MV-FFF with 1,800 MU/min, respectively. (a) and (b) are based on the plan of Case 1; (c) and (d) from Case 2. (1%/1 mm gamma criterion (10% low-dose threshold))

Based on the calculated dose of the original and modified plans with simulated delivery errors, the dose difference for each organ structures was compared. The dosimetric influence of the delivery error occurring at the inter-segment breakpoint was investigated, as summarized in Table S.D.3 and S.D.4. For targets that were mainly located in the isocenter, the dose difference owing to the change in deliverability by plan parameters was up to 0.21 %. Conversely, for OAR mainly located off-isocenter, the dose differences were non-negligible, up to -8.77%.

|  |  | Number of control points | | | | | | | | | | | | | | | | | |
| --- | --- | --- | --- | --- | --- | --- | --- | --- | --- | --- | --- | --- | --- | --- | --- | --- | --- | --- | --- |
|  |  | 25 | | | 49 | | | 73 | | | 91 | | | 145 | | | 241 | | |
|  |  | Original (Gy) | Modified (Gy) | Diff  (%) | Original (Gy) | Modified (Gy) | Diff  (%) | Original (Gy) | Modified (Gy) | Diff  (%) | Original (Gy) | Modified (Gy) | Diff  (%) | Original (Gy) | Modified (Gy) | Diff  (%) | Original (Gy) | Modified (Gy) | Diff  (%) |
| Case 1  (6 MV-FFF) | PTV | 43.770 | 43.863 | 0.21 | 43.734 | 43.747 | 0.03 | 43.699 | 43.694 | -0.01 | 43.631 | 43.639 | 0.02 | 43.622 | 43.624 | 0 | 43.954 | 43.952 | 0 |
|  | Lung_Lt | 0.720 | 0.716 | -0.56 | 0.734 | 0.729 | -0.68 | 0.735 | 0.731 | -0.54 | 0.718 | 0.715 | -0.42 | 0.738 | 0.737 | -0.14 | 0.743 | 0.742 | -0.13 |
|  | Lung_Rt | 1.167 | 1.176 | 0.77 | 1.139 | 1.142 | 0.26 | 1.159 | 1.161 | 0.17 | 1.151 | 1.153 | 0.17 | 1.161 | 1.161 | 0 | 1.184 | 1.185 | 0.08 |
|  | Heart | 0.120 | 0.120 | 0 | 0.119 | 0.119 | 0 | 0.123 | 0.123 | 0 | 0.121 | 0.121 | 0 | 0.124 | 0.124 | 0 | 0.124 | 0.124 | 0 |
|  | Esophagus | 0.553 | 0.553 | 0 | 0.598 | 0.598 | 0 | 0.595 | 0.595 | 0 | 0.580 | 0.580 | 0 | 0.587 | 0.587 | 0 | 0.601 | 0.601 | 0 |
|  | | | | | | | | | | | | | | | | | | | |
| Case 2  (10 MV-FFF) | PTV | 52.303 | 52.383 | 0.15 | 52.243 | 52.188 | -0.11 | 50.982 | 50.953 | -0.06 | 51.157 | 51.185 | 0.05 | 51.453 | 51.463 | 0.02 | 51.081 | 51.071 | -0.02 |
|  | Liver | 5.427 | 5.449 | 0.41 | 5.385 | 5.379 | -0.11 | 5.406 | 5.399 | -0.13 | 5.140 | 5.142 | 0.04 | 5.481 | 5.482 | 0.02 | 5.434 | 5.432 | -0.04 |
|  | Stomach | 1.284 | 1.232 | -4.05 | 1.316 | 1.320 | 0.30 | 1.354 | 1.355 | 0.07 | 1.186 | 1.186 | 0 | 1.364 | 1.364 | 0 | 1.360 | 1.356 | -0.29 |
|  | Heart | 0.071 | 0.073 | 2.82 | 0.071 | 0.071 | 0 | 0.112 | 0.112 | 0 | 0.059 | 0.059 | 0 | 0.113 | 0.113 | 0 | 0.109 | 0.109 | 0 |
|  | Esophagus | 1.243 | 1.134 | -8.77 | 1.285 | 1.288 | 0.23 | 1.306 | 1.307 | 0.08 | 1.160 | 1.161 | 0.09 | 1.281 | 1.282 | 0.08 | 1.326 | 1.325 | -0.08 |

**Table S.D.3.** Dose differences by target and OAR between original and modified plan with simulated delivery errors, according to different numbers of control points

|  |  | Dose rate (MU/min) | | | | | | | | | |
| --- | --- | --- | --- | --- | --- | --- | --- | --- | --- | --- | --- |
|  |  | 600 | | | 1400 | | | 1800 | | | |
|  |  | Original (Gy) | Modified (Gy) | Diff  (%) | Original (Gy) | Modified (Gy) | Diff  (%) | Original (Gy) | Modified (Gy) | Diff  (%) |  |
| Case 1 (6 MV-FFF) | PTV | 40.966 | 40.991 | 0.06 | 43.770 | 43.863 | 0.21 |  | | |  |
|  | Lung_Lt | 0.755 | 0.753 | -0.26 | 0.720 | 0.716 | -0.56 |  |  |  |  |
|  | Lung_Rt | 1.221 | 1.223 | 0.16 | 1.167 | 1.176 | 0.77 |  |  |  |  |
|  | Heart | 0.180 | 0.180 | 0 | 0.120 | 0.120 | 0 |  |  |  |  |
|  | Esophagus | 0.579 | 0.579 | 0 | 0.553 | 0.553 | 0 |  |  |  |  |
|  | | | | | | | | | | | |
| Case 2  (10 MV-FFF) | PTV |  | | | 46.581 | 46.622 | 0.09 | 52.303 | 52.383 | 0.15 |  |
|  | Liver |  |  |  | 4.767 | 4.778 | 0.23 | 5.427 | 5.449 | 0.41 |  |
|  | Stomach |  |  |  | 1.113 | 1.088 | -2.25 | 1.284 | 1.232 | -4.05 |  |
|  | Heart |  |  |  | 0.067 | 0.068 | 1.49 | 0.071 | 0.073 | 2.82 |  |
|  | Esophagus |  |  |  | 1.061 | 1.011 | -4.71 | 1.243 | 1.134 | -8.77 |  |

**Table S.D.4.** Dose differences by target and OAR between original and modified plan with simulated delivery errors, according to different dose rates
